# Psychosocial determinants of consistent condom use among university students in Sudan: findings from a study using the Integrated Change Model

**DOI:** 10.1186/s12889-023-15466-5

**Published:** 2023-03-28

**Authors:** Husameddin Farouk Elshiekh, Ciska Hoving, Hein de Vries

**Affiliations:** CAPHRI School for Public Health and Primary Care, Department of Health Promotion, Maastricht University, P.O. Box 616, Maastricht, 6200 MD the Netherlands

**Keywords:** Sudan, HIV, Condom use, University students, I-Change model

## Abstract

Unprotected sex is common among university students in Sudan, thus increasing risks for sexually transmitted diseases (STDs) and human immunodeficiency virus (HIV). As little is known about the psychosocial determinants of consistent condom use among this population, this study was designed to identify them. The Integrated Change Model (ICM) was applied in a cross-sectional design to identify in 218 students (aged 18–25 years) from Khartoum which items distinguish condom users from non-condom users. Condom users differed significantly from non-condom users in having more HIV and condom use-related knowledge, higher perception of susceptibility to HIV, reporting more exposure to condom use cues, having a less negative attitude towards condom use (attitude cons), experiencing social support and norms favouring condom use and having higher condom use self-efficacy. Binary logistic regression showed that peer norms favouring condom use in addition to HIV-related knowledge, condom use cues, negative attitude and self-efficacy were the factors uniquely associated with consistent condom use among university students in Sudan. Interventions seeking to promote consistent condom use among sexually active students could benefit from increasing knowledge about HIV transmission and prevention, raising HIV-risk perception, using condom use cues, addressing perceived condom disadvantages and enhancing students` self-efficacy to avoid unprotected sex. Moreover, such interventions should raise students` perceptions of their peers` beliefs and behaviours favouring condom use and seek health care professionals` and religious scholars` support for condom use.

## Introduction

In 2010, the global HIV response aimed to achieve three zeros by 2030; zero new HIV infections, zero AIDS-related deaths and zero discrimination against people living with HIV/AIDS (PLWHA). To achieve these goals, measurable targets were set and interim 2020 milestones were articulated by the United Nations (UN) General Assembly in the 2016 Political Declaration on Ending AIDS. These milestones included reducing new HIV infections to fewer than 500,000 by 2020 through ensuring access to combination prevention options, including pre-exposure prophylaxis, voluntary medical male circumcision, harm reduction and condom promotion. However, the progress towards these goals is off track in the Middle East and North Africa (MENA), where HIV infections have increased by 22% since 2010 [1]. Sudan is among the countries with the highest HIV prevalence in MENA. In 2013, it was estimated that around 21% of PLWHA in MENA were from Sudan [2].

Sudan has a total population of about 35 million people. The number of people living with HIV in Sudan in 2019 was estimated at 46,000 and the estimated HIV prevalence was 0.2% [3]. Although this is considered a low prevalence compared to the generalized epidemic in some sub-Saharan countries such as South Sudan (2.5%) [4], the prevailing lack of knowledge, increasing poverty and political instability in the country raise the concerns as these conditions could further fuel the epidemic in Sudan [5]. The HIV epidemic in Sudan is spread mainly via unprotected heterosexual and among men who have sex with men [6]. In Sudan, all types of extramarital sex are religiously forbidden and socially unaccepted; social norms expect sexual abstinence and virginity at the time of marriage remains a virtue. Therefore, condom promotion programs are difficult to implement in the country and previous attempts to promote condom use among university students were resisted by the religious leaders who believe that condom promotion will promote immorality and promiscuity.

University students are targeted by HIV prevention interventions in many countries because they are believed to be at a higher risk of acquiring HIV compared to the general population in these countries [7–9]. This has also been observed in many countries in the MENA region, where increasing numbers of university students become involved in risky sexual behaviours [10–12]. Engagement of university students in sexual risk behaviours can be attributed to several factors, including the university lifestyle with diminished parental control and monitoring and the poor comprehensive knowledge about HIV/AIDS [13–15]. In addition, poor access to HIV counselling and sexual health services is also a determinant of high-risk sexual behaviours among this population [16]. Alcohol and drug use among university students are also associated with increased high-risk behaviours, including unprotected sex with multiple sex partners [16, 17].

Similarly, university students in Sudan are at high risk of contracting HIV because of a relatively high prevalence of engagement in condomless sex. Based on a survey conducted by the Sudan National AIDS Program (SNAP) in 2002, it was estimated that about 6.5% of university students in Sudan were sexually active (*unpublished report*). In 2010, a study conducted by SNAP among higher education institutions` students and staff in Sudan revealed an increase in sexual activity among university students to more than 12.5%. Moreover, only 20% of the sampled university students reported using condoms during their first-ever sexual encounter and only 32% of them used a condom during their latest sexual intercourse (*unpublished report*). In another study conducted among visitors to voluntary counselling and testing (VCT) centers in Khartoum, only 12% of the respondents reported using condoms consistently, 41.5% used them sporadically and 46.3% were nonusers. According to this study, knowledge about AIDS transmission, knowing someone infected with or had died of AIDS, experiencing condom problems, type of sexual partners, purchase embarrassment and education were the main predictors of condom use [18]. However, this study included only male participants.

Considering the importance of the psychosocial determinants of condom use, several behavioural change theories have been used to promote condom use through addressing these determinants [19, 20]. The Integrated Change Model (I-Change Model) is one of these theories used to explain a variety of types of health behaviour, including consistent condom use [21, 22]. The I-Change Model, which is derived from the Attitude – Social influence – Self-Efficacy Model, integrates the ideas of Ajzen’s Theory of Planned Behavior, Bandura’s Social Cognitive Theory, Prochaska’s Transtheoretical Model, the Health Belief Model, and goal setting theories [23]. The I-Change Model distinguishes three phases in the process of behavioural change: a pre-motivational (awareness), motivational and post-motivational (action) phase. Each of these three phases has its relevant determinants [24]. The pre-motivational or awareness phase is determined by knowledge, risk perceptions, cues to action and cognisance about one`s own behaviour. In relation to this study, the model assumes that condom use pre-motivational awareness phase is determined by a person`s cognisance of his/her sexual behaviour and whether it meets the recommendations, accurate knowledge about HIV and condom use, and a person’s perception of the seriousness of HIV (risk severity) and how likely it is to get HIV if practised condomless sex (risk susceptibility). This phase is also determined by the cues that prompt a person to use condoms consistently such as the death of a relative with AIDS. Once they become aware of the health problem (HIV) and its risk behaviours (condomless sex), individuals can proceed to the motivational phase in which they will consider taking up a health-promoting behaviour (e.g. consistent condom use). According to the I-Change Model, the determinants of this motivational phase include attitude, social influence and self-efficacy. In relation to this study, a person`s attitude towards condom use is his or her perception of the cognitive and emotional advantages and disadvantages of using condoms consistently [21]. The social influence on an individual`s condom use behaviour refers to the support that he or she receives from others to use condoms (social support), the perception of what others in his community believe about condom use (social norm) and the individual`s perception of condom use behaviour among the community members (social modelling) [25]. Self-efficacy refers to a person’s perception of his capability to carry out a type of behaviour (consistent condom use) in a variety of situations and how difficult a person regards realising the desired healthy behaviour [26]. These motivational factors together are assumed to predict the intention to use condoms consistently. The translation of this intention into behaviour is the third and post-motivational phase which is determined by a person’s level of intention, action plans such as the plans required to prepare oneself and initiate condom use and the coping plans needed to overcome barriers and plan enactment. This phase is also determined by a person`s self-efficacy, skills and the level of barriers that are encountered [27]. Finally, as a psycho-social-ecological model, the I-Change model indicates that these factors are influenced by predisposing factors such as psychological factors (e.g. personality), behavioural factors (e.g. lifestyles), social and cultural factors (e.g. policies, cultural norms, religion), biological factors (e.g. gender, genetic predisposition) and information factors (the quality of messages, channels and sources used) [21].

The above-described determinants have been poorly studied in Sudan. To the best of our knowledge, these determinants have been only recently explored by a qualitative study we have conducted among university students in Khartoum using the I-Change Model. Regarding the pre-motivational determinants, the study revealed several misconceptions about condoms and their use among male and female students and most of the participants reported a lack of knowledge about how to use condoms. Regarding risk perception, most of the participants perceived the high risk of getting HIV if they practised condomless sex. They also reported a high perception of HIV severity and indicated that HIV is a serious disease with severe impacts on health and social life. The cues reported by the consistent condom users as encouraging cues included having previous experience with PLWHA and having easy access to condoms. Concerning the motivational determinants, the findings suggested that negative attitude was a determinant of condom use as non-condom users of both sexes perceived several physical and emotional disadvantages associated with condom use. Regarding the role of social influence on the students’ condom use, the study suggested that lack of social support was a barrier and pointed to the role of religious values and social norms against condom use. Most of the participants also pointed to the influential role of their peers in their condom use behaviour. Low self-efficacy was also identified as a possible determinant of condom use as most of the consistent users reported higher confidence in their ability to overcome the challenging situation than non-condom users. Finally, regarding the post-motivational determinants, the study suggested that poor action planning was a barrier as most of the participants reported a lack of action and coping plans [14].

**Fig. 1:**
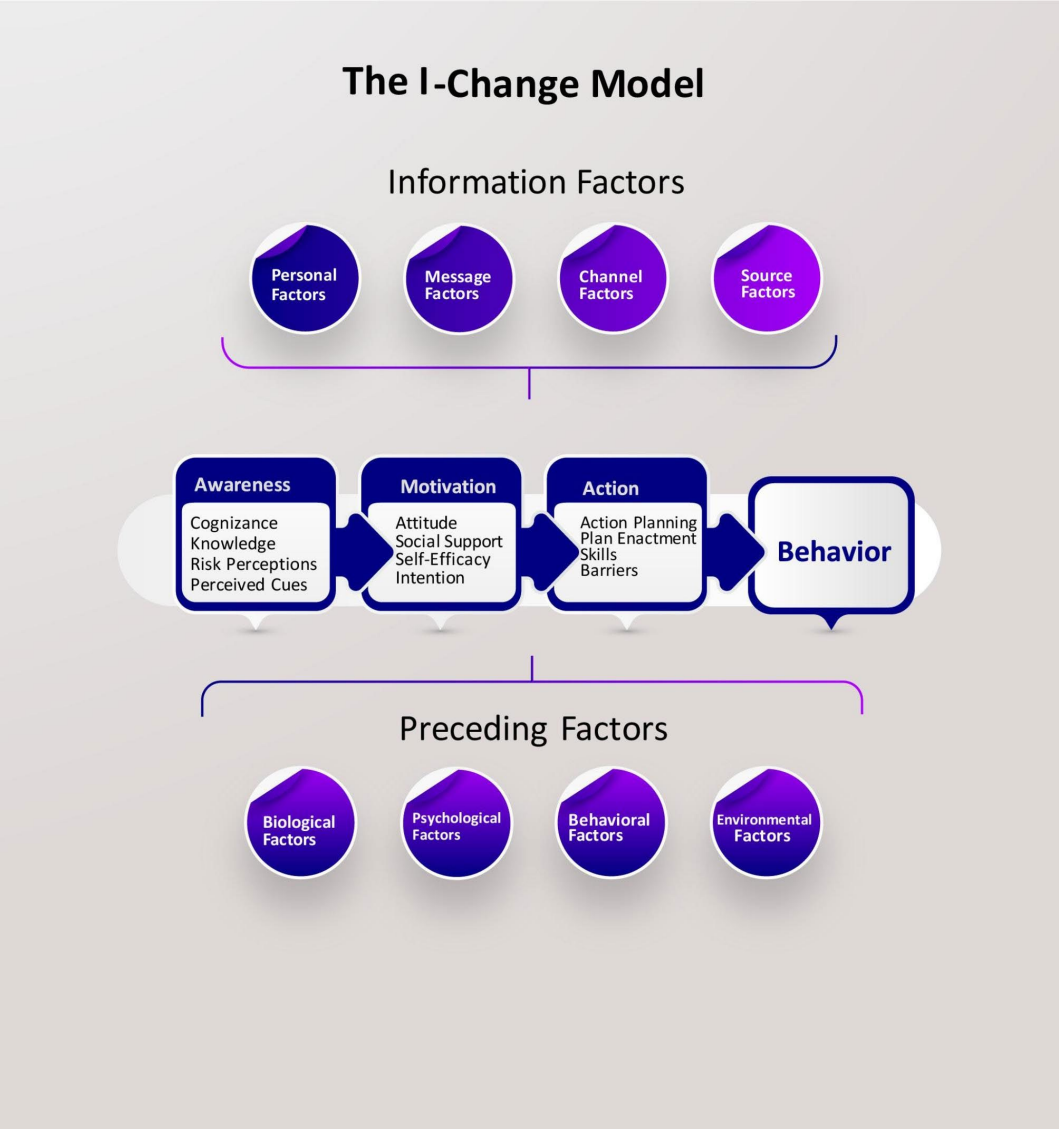
The integrated behavioural change (I-CHANGE) model

Although the previous study provided important insights into the psychosocial determinants of consistent condom use among university students in Sudan, the study was limited by its qualitative design and the small sample of only 30 students. Therefore, this study was conducted to complement the previous study and further assess these determinants quantitatively. For this purpose, the I-CHANGE model (Fig. 1) was used as a theoretical framework.

## Method

### Design

A quantitative cross-sectional study was used among university students in Khartoum.

### Recruitment and participant selection

The target group of this study was the sexually active undergraduate university students in Khartoum state. From a list of 35 universities in Khartoum, including 16 public and 19 private universities, three public and three private universities were randomly selected. The deans of students` affairs in these selected universities were visited by the principal researcher to explain the objectives of the study and seek their approval. Following approvals, invitation letters were distributed among the students in randomly selected lecture rooms in each university. The invitation letters provided to the students described the study and its objectives and shared how to access the study questionnaire online. Additionally, some sexually active students identified by the HIV counsellors were asked to invite their sexually active friends, who were also university students, to fill the online questionnaire (snowball recruitment).

### Procedures

As it is culturally sensitive to talk about sexual behaviour outside marriage in Sudan openly, data collection occurred online. The online questionnaire was in Arabic and accessible by smartphones, laptops and computers. The questionnaire started with an introduction presenting the study and explaining its objectives. This was followed by a section to inform the students that their participation was voluntary, and their confidentiality and privacy were assured. Participants’ identifiers such as their names, address, phone numbers or universities were not included in the questionnaire.

### Measurement

Questionnaire development was inspired using findings of an earlier qualitative study [14] and previous studies about sexual health behaviours using the I-Change Model [27, 28]. The instrument was piloted with ten university students other than those who participated in the study; no extensive changes were required. To assess the validity of the questionnaire for each construct of the I-Change model, factor analysis was conducted. Cronbach’s alpha was calculated to ensure the internal consistency of each construct items [29].

#### Knowledge

Knowledge was assessed by 16 statements about HIV, its transmission, prevention and treatment and five statements about condom use such as “*condoms have expiry dates*” and “*condoms could affect the muscles of the penis*”. Participants could respond to each statement with *yes*, *no* or *not sure*. Participants` responses to knowledge questions were coded as (1) for correct answers and (0) for incorrect or not sure responses.

#### Cues to action

Cues to consistent condom use were assessed by five items; knowing someone infected with or died of HIV, previously attending a talk regarding living with HIV/AIDS, previously attending a peer education program about HIV and knowing someone who could provide condoms. Participants could answer with *yes* (1) or *no* (0) for each item. All of these factors were combined together as cues to consistent condom use.

#### Risk perception

Risk perception was assessed by five items. Three items assessed the participants` perception of the risk of severe HIV-related health problems, social problems and psychological distress (-2 (totally disagree) to + 2 (totally agree); Cronbach’s alpha = 0.74). To assess participants` perception of susceptibility to HIV, they were asked how likely they would be infected with HIV if they practised unprotected vaginal sex and how likely they would be infected with HIV if they practised unprotected anal sex (-2 (very unlikely) to + 2 (very likely); Cronbach’s alpha = 0.83).

#### Attitude

To assess attitude towards condom use, five items were used for the advantages (pros) of using condoms such as “*If I use condoms during sexual intercourse, I will be protected against HIV and other STIs”* and another five items for the disadvantages (cons) of using condom such as “*If I use condoms during sexual intercourse, this will indicate that I do not trust my partner.,”* (Cronbach’s alpha = 0.64 and 0.78, respectively). Response options for all attitude items ranged from − 2 (totally disagree) to + 2 (totally agree).

#### Social influence

Social influence was assessed with eleven items such as “*Most of my friends believe that I should use condoms during sexual intercourse”* and “*Most of my sexual partners support me to use condoms during sexual intercourse.”* For all social influence items, participants could reply on a five-point Likert scale ranging from − 2 (totally disagree) to + 2 (totally agree). Based on factor analysis, the social influence 11 items were grouped into three categories: peer norm (two items; Cronbach’s alpha = 0.68), peer support and modelling (three items; Cronbach’s alpha = 0.71) and others (parents, religious scholars and health professional) influence (six items; Cronbach’s alpha = 0.82).

#### Self-efficacy

Self-efficacy was assessed with six statements such as “*I would find it difficult to use condoms if my sexual partners refuse it”*. Participants could reply to self-efficacy items on a five-point Likert scale ranging from − 2 (totally agree) to + 2 (totally disagree). (Cronbach’s alpha = 0.78)

#### Intention

Intention to use condoms consistently was assessed with three statements using a five-point Likert scale regarding respondents’ intention to use condoms during the subsequent sexual intercourse, to use it consistently during future sexual intercourse and to discuss condom use with the sexual partner during the following sexual intercourse (-2 (totally disagree) to + 2 (totally agree); Cronbach’s alpha = 0.84).

#### Consistent condom use

Consistent condom use as a behavioural outcome variable was measured by asking the participants whether they used a condom during their last sexual intercourse (*yes*, *no*) and how frequently they use condoms during sexual intercourse (*always, often, sometimes, rarely, never).* Only those who stated that they used condoms during their last sexual activity and always used condoms during sex were considered consistent condom users. Accordingly, non-consistent condom users were coded as (0) and consistent condom users (1).

### Data analysis

Data analysis was performed in SPSS version 24. A descriptive analysis was undertaken to describe the study sample. Multivariate analysis of variances (MANOVA) was conducted to assess the difference between consistent and non-consistent condom users per psychosocial construct and for each individual construct item. Finally, forward binary logistic regression analysis (forward LR) was performed to identify the potential predictors of consistent condom use. Results with *p* values < 0.05 were considered significant.

## Results

### Description of the sample

Initially, about 415 students responded, but only 304 of them completed the whole questionnaire. The remaining 111 participants answered less than 70% of the questions; therefore, they were excluded from the study. Among those who completed the online questionnaire, 98 were sexually active. An additional 120 sexually active participants were recruited by some sexually active students (snowball recruitment). Two hundred and eighteen sexually active male and female university students were included in the study. The sample included 76 consistent condom users (35%). Most participants were Sudanese (94.5%) and Muslim (94%). Table 1 summarises the demographic characteristics of the study participants.

**Table 1:** Description of the study sample (*N* = 218)

Characteristics		Total N (%)	Non-consistent condom users	Consistent Condom users	*χ* ^*2*^	*P*-value
**Consistent condom use**		218 (100%)	142 (65%)	76 (35%)		
**Age** (range 18–25)	Mean	21.06	21.1	21		
**Age group**						
< 20 years		80 (37%)	50 (62.5%)	30 (37.5%)	0.39	0.53
> 20 years		138 (63%)	92 (66.7%)	46 (33.3%)		
**Gender**						
Male		137 (63%)	93 (67.9%)	44 (32.1%)	1.22	0.27
Female		81 (37%)	49 (60.5%)	32 (39.5%)		
Nationality group*						
Sudanese		206 (94%)	134 (65%)	72 (35%)		1.0
Non-Sudanese		12 (6%)	8 (66.7%)	4 (33.3%)		
Religious group*						
Muslims		205 (94%)	131 (63.9%)	74 (36.1%)		0.23
Non-Muslims		13 (6%)	11 (84.6%)	2 (15.4%)		
**Family income**						
Low income		51 (24%)	28 (54.9%)	23 (45.1%)	3.09	0.21
Middle income		99 (45%)	68 (68.7%)	31 (31.3%)		
High income		68 (31%)	46 (67.6%)	22 (32.4%)		
**Type of university**						
Public university		118 (54%)	76 (64.4%)	42 (35.6%)	0.06	0.81
Private university		100 (46%)	66 (66%)	34 (34%)		
Academic year*						
Early academic years (1st − 3rd years)		106 (49%)	66(62%)	40 (38%)	0.75	0.39
Late academic years (4th - 6th years)		112 (51%)	76 (68%)	36 (32%)		

* Fisher`s exact test was used instead of Chi-squared test

### Pre-motivational determinants

#### Knowledge about HIV/AIDS

The overall percentage of correct answers to each of the 16 items used to assess knowledge about HIV/AIDS ranged between 27 and 85%. The vast majority of the participants correctly answered items on HIV transmission. Nevertheless, misconceptions existed among the students. Many of them believed that HIV could be transmitted by mosquito bites or through hugging people living with HIV/AIDS (PLWHA) (43% and 45% respectively). More than 70% also believed that HIV transmission could be prevented by pre-ejaculation withdrawal. Additionally, more than 60% had the misconception that most people know they are infected with HIV soon after being infected.

MANOVA results showed that the overall knowledge about HIV/AIDS (the total score of all knowledge items) was higher among consistent condom users as compared to non-consistent condom users (Hotelling’s T = 0.282; F (16,201) = 3.539; *p* < .001). When looking at the separate HIV knowledge items, significant differences were observed between consistent and non-consistent condom users with higher knowledge about HIV/AIDS among consistent condom users in almost all items, as shown in Table 2.

**Table 2:** Differences between groups for knowledge and cues

Knowledge about HIV/AIDS	Overall mean	Non- consistent condom users	Consistent Condom users	*F*	*P*
Anyone can get infected with HIV if he practices condomless vaginal intercourse with infected persons	0.83	0.75	0.96	15.66	**< 0.001**
Anyone can get infected with HIV if he practices condomless anal intercourse with infected persons	0.78	0.72	0.91	10.95	**< 0.01**
Anyone can get infected by getting injections with a needle that has already been used by infected persons.	0.84	0.81	0.89	2.65	0.105
A pregnant woman who is infected with HIV can transmit the virus to her baby.	0.71	0.65	0.80	5.29	**< 0.05**
Anyone can get infected with HIV from a mosquito bite.	0.53	0.47	0.63	5.14	**< 0.05**
Anyone can get infected with HIV through hugging with people living with HIV.	0.55	0.44	0.75	21.35	**< 0.001**
Someone who looks healthy can be infected with HIV.	0.68	0.63	0.78	5.15	**< 0.05**
You can protect yourself against HIV by abstaining from sexual intercourse before marriage.	0.85	0.82	0.91	3.21	0.075
You can protect yourself against HIV by using a condom correctly every time you have sexual intercourse.	77	0.68	0.95	22.56	**< 0.001**
People can reduce the risk of getting HIV by reducing the number of their sexual partners.	0.66	0.56	0.83	16.50	**< 0.001**
Having sexually transmitted infection put you at higher risk of getting infected with HIV.	0.71	0.63	0.86	12.38	**< 0.01**
Most people do know they are infected with HIV soon after getting infected	0.32	0.26	0.42	6.00	**< 0.05**
Getting the penis out just before ejaculation, is a safe method of preventing HIV transmission	0.27	0.26	0.28	0.06	0.803
HIV treatments help HIV infected people to live normally for longer time.	0.61	0.56	0.71	5.02	**< 0.05**
HIV infected people on treatment are less likely to transmit HIV to others.	0.56	0.47	0.74	14.98	**< 0.001**
Early diagnosis of HIV infection can prevent development of AIDS.	0.66	0.62	0.72	2.38	0.125
**Knowledge about condom use**					
Condoms could affect the muscles of the penis	0.37	0.29	0.53	12.55	**< 0.01**
Condoms protect only male partner against HIV and sexually transmitted infections (STI)	0.61	0.57	0.68	2.70	0.102
Consistent use of condoms can cause loss of sexual desire.	0.29	0.20	0.46	16.74	**< 0.001**
Consistent condom use provides only 50% protection against HIV	0.41	0.32	0.58	14.01	**< 0.001**
Condoms have expiry dates	0.80	0.75	0.91	8.39	**< 0.01**
**Condom use cues**					
Do you know someone who is infected with HIV/AIDS	0.18	0.13	0.26	5.74	**< 0.05**
Do you know someone who died of AIDS	0.14	0.10	0.21	5.31	**< 0.05**
Have you ever listened to someone living with HIV/AIDS telling his experience of living with HIV	0.21	0.11	0.39	26.29	**< 0.001**
Have you attended any peer-education program on HIV prevention	0.31	0.17	0.58	46.71	**< 0.001**
Do you know somebody who can provide you with condoms confidentially	0.36	0.15	0.75	115.83	**< 0.001**

Knowledge items: *(correct = 1, incorrect = 0)*, Cues items: *(yes = 1, no = 0)*

#### Knowledge about condom use

The results reveal several misconceptions about condoms and their use prevalent among the total study population. For example, the majority of the students believed that consistent condom use could affect the muscles of the penis and cause loss of sexual desire (68% and 73%, respectively). In addition, about 59% also underestimated the protective role of consistent condom use against HIV transmission. Besides, 39% of the participants believed that condoms protect only male partners against HIV and sexually transmitted infections (STI).

When comparing consistent and non-consistent condom users using MANOVA, overall knowledge about condom use was higher among consistent condom users (Hotelling’s T = 0.163; *F* (5,212) = 6.927; *p* < .001) with significantly higher knowledge about condom use among consistent condom users in almost all items (Table 2).

#### Cues to action

The participants reported low exposure to the cues about condom use. Less than 20% of them knew someone infected or who had died of HIV/AIDS. Only 31% had attended a peer education program about HIV prevention and only 36% of them knew somebody who could provide them with condoms confidentially. Generally, consistent condom users reported higher exposure to condom use cues than non-consistent condom users (Hotelling’s T = 0.550; *F* (5,212) = 23.307; *p* < .001). When looking at the items separately, consistent condom users had significantly higher exposure to all of these cues than non-consistent users (Table 2).

#### Risk perception

The participants generally had high perceptions of the severe health, social and psychological consequences of HIV infection. MANOVA showed no overall difference between the two groups in their perception of HIV severity (Hotteling`s T = 0.019; *F* (3,214) = 1.382; *p* = .249). In contrast, the perception of susceptibility to HIV was relatively low among the study participants; however, consistent condom users scored significantly higher to the overall perception of susceptibility than non-consistent condom users (Hotelling’s T = 0.086; *F* (5,212) = 9.245; *p* < .001). In addition, compared to non-consistent condom users, consistent users showed higher perceptions of susceptibility to HIV if they practised unprotected vaginal (*p* < .01) and anal sex (*p* < .001), as shown in Table 3.

**Table 3:** Differences between groups for HIV risk perception, attitude, social influence, self-efficacy and intention

Construct items	Overall mean	Non- consistent condom users (Mean score)	Consistent Condom users (Mean score)	*F*	*P*
**Risk perception (Risk severity)**					
If I would contract HIV, this would be a serious health problem for me.	1.58	1.51	1.72	4.090	**< 0.05**
If I would contract HIV, I would have serious social problems.	1.59	1.54	1.67	1.430	0.233
If I would contract HIV, I would suffer from serious psychological distress.	1.51	1.46	1.59	1.022	0.313
**Risk perception (Risk susceptibility)**					
How likely that you will get HIV infection if you practice unprotected vaginal intercourse.	0.45	0.30	0.72	6.883	**< 0.01**
How likely that you will get HIV infection if you practice unprotected anal intercourse.	0.43	0.18	0.88	18.294	**< 0.001**
**Attitude (pros condom use)** If I use condoms during sexual intercourse:					
I will be protected against HIV and other STIs	1.24	1.23	1.26	0.102	0.750
This will indicate that I care about my partner`s health.	1.15	1.18	1.11	0.268	0.605
I don’t have to worry about pregnancy	1.32	1.37	1.21	1.707	0.193
This will help me to have more sexual partners.	− 0.05	− 0.14	0.12	2.557	0.111
It will delay ejaculation and let me enjoy sex	0.66	0.63	0.72	0.517	0.473
**Attitude (cons condom use)** If I use condoms during sexual intercourse:					
This will indicate that I do not trust my partner.	− 0.42	− 0.32	− 0.61	2.737	0.100
It will decrease sexual pleasure	0.22	0.77	− 0.80	114.946	**< 0.001**
I will have health problems due to semen stagnation	0.13	0.23	− 0.05	3.573	0.060
I will become sex addicted	0.47	0.62	0.18	9.182	**< 0.01**
It will feel unnatural to me	− 0.51	− 0.43	− 0.66	1.923	0.167
**Social influence (Peer norm)**					
Most of my friends believe that I should use condoms during sexual intercourse.	0.27	0.16	0.47	3.507	0.062
Most of my sexual partners believe that I should use condoms during sexual intercourse	− 0.08	− 0.18	0.12	3.780	0.053
**Social influence (Peer support & modelling)**					
Most of my friends support me to use condoms during sexual intercourse	0.18	− 0.05	0.62	15.652	**< 0.001**
Most of my sexual partners support me to use condoms during sexual intercourse	− 0.16	− 0.38	0.25	15.986	**< 0.001**
How many of your friends use condoms during sexual intercourse?	− 0.15	− 0.30	0.12	8.282	**< 0.01**
**Social influence (Others influence)**					
My parents believe that I should use condoms during sexual intercourse	0.32	0.30	0.36	0.113	0.737
Health professionals believe that I should use condoms during sexual intercourse	0.85	0.73	1.09	9.676	**< 0.01**
Religious scholars believe that I should use condoms during sexual intercourse.	0.41	0.27	0.67	7.209	**< 0.01**
My parents support me to use condoms during sexual intercourse.	0.37	0.32	0.45	0.702	0.403
Health professionals support me to use condoms during sexual intercourse.	0.85	0.68	1.16	15.771	**< 0.001**
Religious scholars support me to use condoms during sexual intercourse.	0.39	0.25	0.64	7.429	**< 0.01**
**Self-efficacy**					
I would find it difficult to use condoms if my sexual partners refuse it.	− 0.76	− 0.91	− 0.47	9.325	**< 0.01**
I would find it difficult to use condoms in case of high sexual arousal.	− 0.38	− 0.65	0.13	23.447	**< 0.001**
I would find it difficult to use condoms because it is difficult for me to get it.	− 0.50	− 0.68	− 0.17	10.180	**< 0.01**
I would find it difficult to use condoms when I feel that it reduces pleasure.	− 0.44	− 0.63	− 0.09	11.651	**< 0.01**
I would find it difficult to use condoms with my steady partner.	− 0.64	− 0.72	− 0.48	2.019	0.157
I would find it difficult to use condoms since I do not know how to use it properly.	0.27	0.06	0.66	16.490	**< 0.001**
**Intentions**					
I have the intention to use condoms during the next sexual intercourse	0.93	0.80	1.17	8.763	**< 0.01**
I have the intention to use condoms consistently during future sexual intercourse	0.86	0.73	1.11	9.002	**< 0.01**
I have the intention to discuss condoms use with my sexual partner the next time I have sex	1.06	0.89	1.38	16.771	**< 0.001**

Risk perception (severity), Attitude, social influence and intentions items: *(-2 (totally disagree) to + 2 (totally agree))* Risk perception (susceptibility) items: *(-2 (very unlikely) to + 2 (very likely));* Self-efficacy items: *(-2 (totally agree) to + 2 (totally disagree)*

### Motivational determinants

#### Attitude

The comparison between consistent and non-consistent condom users in their perception of the advantages of using condoms revealed no significant difference (Hotteling`s T = 0.026; *F* (5,212) = 1.121; *p* = .350). Among the items that assessed the positive attitude towards condom use (condom use pros), protection against pregnancy, prevention of HIV/STIs and indicating caring about partner’s health were the most important perceived advantages of consistent condom use among the study population.

On the other hand, a high perception of condom use disadvantages (condom use cons) was observed, with a significantly higher perception of condom use disadvantages among non-consistent users (Hotteling`s T = 0.608; *F* (5,212) = 25.795; *p*< .001). For instance, non-consistent condom users were more convinced than consistent condom users that condom use would decrease sexual pleasure (*p* < .001) and lead to sex addiction (*p* < .01). The perceptions that consistent condom use would indicate lack of trust in the sexual partner, cause semen stagnation or feel unnatural were all very low among both consistent and non-consistent condom users with no significant differences between the two groups (Table 3).

#### Social influence

Generally, the participants had a very low perception of peer norms favouring consistent condom use. Consistent condom users had a relatively higher perception of friends and sexual partners` norms favouring consistent condom use; however, this difference was not statistically significant (p = .062 and 0.053, respectively). In addition, no difference in overall peer norm influence was identified between consistent and non-consistent condom users (Hotteling`s T = 0.022; *F* (2,215) = 2.403; *p* = .093). Overall, consistent condom users reported more peers to use and support condom use (Hotteling`s T = 0.102; *F* (3,214) = 7.3; *p* < .001). In-depth analysis revealed that most of the participants received little support from their friends and sex partners, but consistent condom users did report relatively more support from their friends (*p* < .001) and sexual partners (*p* < .001). They also believed that condoms were more commonly used by their peers as compared to non-consistent condom users (*p* < .01).

Consistent condom users also reported to experience more influence from parents, religious leaders and health professionals than non-consistent condom users (Hotteling`s T = 0.111; *F* (6,211) = 3.901; *p* = .001). When looking at the items separately, consistent condom users were more convinced that health professionals (*p* < .01) and religious leaders (*p* < .01) favoured consistent condom use than non-consistent condom users. Consistent condom users also perceived greater support to use condoms consistently from health professionals (*p* < .001) and religious leaders (*p* < .01) than non-consistent condom users. Both groups perceived greater support from health professionals than religious leaders. Parents` influence on consistent condom use, including both parents` support and norms, was not statistically significant (Table 3).

#### Self-efficacy

Students’ self-efficacy to use condoms consistently was generally very low. Partner refusal to use condoms, practising sex with steady partners and facing difficulty in obtaining condoms were the most difficult barriers affecting students` self-efficacy. Overall, consistent condom users reported higher self-efficacy than non- consistent condom users (Hotteling`s T = 0.182; *F* (6,211) = 6.388; *p* < .001). In-depth analysis reveals that consistent condom users showed significantly higher self-efficacy for most difficult situations except when practising sex with steady partners (Table 3).

#### Intention

In general, participants had a slightly positive intention to use condoms during the following sexual intercourse, use it consistently during future sexual intercourse and discuss condom use with their sexual partners the next time they have sex (overall mean scores 0.93, 0.86 and 1.06, respectively). These intentions to use condom were significantly higher for consistent users compared to non-consistent condoms users (Hotteling`s T = 0.081; *F* (3,214) = 5.783; *p* < .01). Consistent condom users had significantly higher intentions to use condoms during the next sexual intercourse (*p* < .01) and to use it consistently during future sexual intercourse (*p* < .01). They also had higher intentions to discuss condom use with their sexual partners the next time they had sex (*p* < .001) (Table 3).

### Regression analysis

Table 4 summarizes the results of the forward binary logistic regression, which showed that HIV knowledge, condom use cues, attitude cons, peer norms and self-efficacy were all uniquely associated with consistent condom use. The odds of consistent condom use were higher among those with higher HIV knowledge (OR: 1.27, 95% CI: 1.22, 144, *p* < .001), higher exposure to condom use cues (OR: 1.74, 95% CI: 1.38, 2.19, *p* < .001) and higher perception of peer norms favoring consistent condom use (OR: 1.65, 95% CI: 1.099, 2.47, *p* < .05). Conversely, the odds of consistent condom use were much lower among those with a higher perception of condom use disadvantages (attitude cons) (OR: 0.15, 95% CI: 0.07, 0.32, *p* < .001). Self-efficacy was found to be strongly associated with consistent condom use. The odds of consistent condom use were more than two times more among those with higher self-efficacy (OR: 2.115, CI: 1.255, 3.566, *p* = .005). The logistic regression model was statistically significant, χ2 (5, N = 218) = 111.691, *p* < .001. The model explained 55.3% (Nagelkerke R^2^ ) of the variance in consistent condom use and correctly classified 81.2% of cases.

**Table 4:** Binary logistic regression analysis for condom use

Variables in the equation			Odds ratio	95% confidence interval		Sig.
				Lower	Upper	
BLOCK 2	Step 1	Condom use cues	1.822	1.498	2.216	**< 0.001**
	Step 2	Knowledge about HIV/AIDS	1.281	1.143	1.436	**< 0.001**
		Condom use cues	1.747	1.422	2.147	**< 0.001**
BLOCK 3	Step 1	Knowledge about HIV/AIDS	1.282	1.135	1.449	**< 0.001**
		Condom use cues	1.789	1.432	2.237	**< 0.001**
		Attitude cons	0.198	0.101	0.386	**< 0.001**
	Step 2	Knowledge about HIV/AIDS	1.278	1.130	1.446	**< 0.001**
		Condom use cues	1.708	1.362	2.142	**< 0.001**
		Attitude cons	0.181	0.089	0.368	**< 0.001**
		Self-efficacy	2.188	1.308	3.660	**< 0.01**
	Step 3	Knowledge about HIV/AIDS	1.271	1.122	1.440	**< 0.001**
		Condom use cues	1.738	1.378	2.191	**< 0.001**
		Attitude cons	0.150	0.070	0.320	**< 0.001**
		Peer norm	1.648	1.099	2.470	**< 0.05**
		Self-efficacy	2.115	1.255	3.566	**< 0.01**

*In block 1, both included variables (age and gender) were not retained in the equation

## Discussion

This study aimed to identify the psychosocial determinants of condom use among university students in Khartoum, using the I-Change Model as a theoretical framework. The findings of the analyses of variance clearly indicated that condom users differed significantly from non-condom users in having more HIV and condom use-related knowledge, higher perception of susceptibility to HIV, reporting more exposure to condom use cues, having a less negative attitude towards condom use (attitude cons), experiencing social support and norms favouring condom use and having higher condom use self-efficacy. These outcomes suggest that, in order to promote condom use, these items should be clearly addressed in condom promotion programs among this at-risk population in Sudan.

The results of the regression analysis also supported the importance of knowledge about HIV/AIDS as a factor uniquely related to consistent condom use among the study population, a finding consistent with results of previous studies [18, 30–32]. Despite being university students, serious knowledge gaps and misconceptions about HIV transmission and prevention as well as condom use misconceptions were revealed by this study. Hence, it is important to design health education messages to address these misconceptions and fill the knowledge gaps. However, holding mass educational campaigns to promote condom use among university students in Sudan is challenging. A recent study has also identified peers as the main source of knowledge about HIV and condom use for university students in Sudan [14]. Therefore, it is essential to select the most appropriate channels to deliver these messages to disseminate HIV knowledge among the students.

The perception of HIV severity was not associated with consistent condom use among this study participants, which contradicts the findings of some previous studies [33–35]. This lack of association could be explained by the high level of social stigma associated with HIV in Sudan that led all the students to perceive the severe social and psychological consequences of contracting HIV as observed in this study and previously reported [14]. However, an association between the perception of susceptibility to HIV and consistent condom use was revealed by this study as well as several previous studies [35–37]. The regression analysis of our study data showed no unique association between HIV-risk perception and consistent condom use although a previous study showed that the influence of risk perception as a pre-motivational factor on behaviour may be mediated by motivational factors as assumed by the I-Change model [38]. Previous match-mismatch studies indicated that people in the pre-motivational phase benefit more from interventions that target their current motivational status [39, 40]. Therefore, it seems important to address HIV-risk perception in condom promotion intervention to raise the awareness of those in the pre-motivational phase.

Regarding the cues to condom use and similar to what was observed in some previous studies [14, 18, 41], knowing someone who was infected with HIV or who died of AIDS was associated with consistent condom use. However, some conditions need to be considered before including this cue in future interventions aiming to promote consistent condom use. Firstly, students’ exposure to such cues may be limited since HIV infected persons in Sudan tend to hide their infection due to the high social stigma and discrimination against PLWHA [42]. Secondly, it has been suggested that fear appeal messages may increase the stigma and discrimination against PLWHA [43]. Besides, previous research suggested that using fear appeal to change the high-risk behaviours among people with low self-efficacy may result in a defensive behaviour to avoid the fear appeal messages [44]. Our study also found an association between knowing somebody who could provide condoms confidentially and consistent condom use, which was also found by previous studies that identified purchase embarrassment as a barrier to consistent condom use [18, 45, 46]. In a conservative community like Sudan, purchasing condoms is usually associated with embarrassment because of the social stigma associated with premarital sex. To cope with this embarrassment, some sexually active students used to ask someone they knew to buy condoms for them or go to pharmacies in remote areas to purchase condoms [14, 18].

Concerning the attitude towards condom use, our study revealed that the participants’ perception of condom disadvantages (cons) was uniquely associated with consistent condom use. Similar to previous research [47, 48], the perceived negative effect of condom use on sexual pleasure was associated with inconsistent condom use among this study population. It should be acknowledged that latex condoms represent mechanical barriers that reduce sensation and physical contact, which could affect sexual pleasure and this represents an important barrier to consistent condom use [47, 49]. However, the effect of condoms on sexual pleasure could be minimised by promoting the use of high-quality condoms and emphasising the pleasure-enhancing aspects of condom use [47].

Regarding social influence on condom use, our study highlighted some important contextual differences between the Islamic and non-Islamic communities that should be considered. According to our study participants, parents` norms and support seemed to play no role in condom use in Sudan due to the Islamic religious values and prevailing social norms prohibiting all types of extramarital sex and discouraging open discussions about sex among family members [14]. This finding contradicts with results obtained from study findings from some non-Islamic cultures [50–52], but maybe explained by the fact that our participants were university students and thus have or want to become less dependent from their dependents. More qualitative in-depth research on this matter may be wanted to understand this finding better. Our study pointed to an interesting result regarding the positive role of Islamic religious scholars in promoting and supporting condom use by sexually active students. Again, this result is not in line with findings from previous studies identifying Islamic religious leaders as opponents to condom use [14, 53]. Nevertheless, other studies also revealed that religious scholars might support condom use by sexually active Muslims considering the Islamic values of preventing harm and disease [54, 55]. Although peer support, modelling and norms favouring consistent condom use were all associated with consistent condom use, only peer norms were uniquely associated with consistent condom use in this study. The importance of peer norms favouring condom use was also reported in other studies [56, 57]. A recent study has identified peers as the only social group among this population with whom sexual practices are discussed and recommended their involvement in condom promotion interventions to facilitate their implementation and maximise their benefits [14].

Self-efficacy was identified by the regression analysis as an important factor with a unique association with consistent condom use in this study, which is in agreement with what has been suggested by a recent qualitative study among the same populations [14], and previous studies [58–60]. Condom use self-efficacy is a multidimensional construct [60]. Previous research reported some gender-specific differences in these self-efficacy dimensions [61], which may necessitate further research to identify such differences among this population to facilitate the design of more gender-specific condom promotion interventions.

Although the association between consistent condom use and the awareness about HIV and the perceived severity and susceptibility to HIV was thoroughly assessed in this study, it should be acknowledged that in such a conservative community the fear of getting unwanted pregnancy could be very influential in students’ intentions to use condoms consistently. Therefore, future research should assess this association to inform future interventions aiming to promote consistent condom use.

## Practice implications

Promoting consistent condom use requires comprehensive interventions that address the different barriers. This study identified the salient psychosocial determinants of consistent condom use to be considered in future condom promotion interventions in Sudan and provided some suggestions on how to deliver such interventions.

HIV-related knowledge, especially HIV transmission and prevention, should be raised. The study identified some of the prevalent misconceptions and HIV-knowledge gaps to be targeted. Future research may investigate if videos of HIV infected persons talking about their experience could be used as cues to promote condom use among Sudanese students with high self-efficacy provided that carefully designed non-discriminatory messages are used. Suitable cue -reminders can also be used to complement interventions and augment their effectiveness [62]. Condom promotion interventions should also address the negative perceptions and emphasise the pleasure-enhancing aspects of condom use. This could be achieved by combining both emotional and factual messages [63].

Moreover, interventions should aim to change students` perception of their peers` beliefs (peer norm) and how they behave (modelling) concerning condom use. Norm-based interventions with strategies such as social norms marketing, personalised normative feedback and focus group discussions could be used for this purpose [64]. Finally, enhancing the students` self-efficacy to use condoms consistently is of paramount importance. All dimensions of condom use self-efficacy must be enhanced using the appropriate techniques such as verbal persuasion, condom use skills, condom negotiation and affect regulation skills [65–67].

A recent randomised control trial has shown that internet-based interventions are effective in behavioural change programs targeting HIV risk behaviour, including condomless sex [68]. This approach has several advantages: maintaining participants privacy, message tailoring, reaching the most at-risk population (MARPs) and saving time and resources [69]. Being the first study using an online questionnaire to study sexual behaviours among this population and considering the sensitive data obtained thorough this approach, our study suggested the suitability, feasibility and acceptability of this approach among university students in Sudan. However, RCT studies to investigate the effectiveness of web-based HIV interventions among this population are highly needed.

## Strengths and limitations

This is the first study focusing mainly on the psychosocial determinants of condom use among university students in Sudan. To the best of our knowledge, it is also the first study that used an online questionnaire to collect data about the sensitive issues around sexual practices among youth in the conservative community of Sudan. This was expected to be more comfortable and popular among university students and assumed to enable researchers to collect more valid data. Using a behavioural change theory, the I-Change model, as a theoretical framework for the study is also one of the strengths as this could help understand the students` condom use behaviour and identify its psychosocial determinants.

Despite these strengths, the study is not free of limitations. Firstly, being a cross-sectional study, a cause-effect relationship could not be established. Secondly, some of the participants were recruited through snowball sampling, which may question the representativeness of the sample and generalizability of the study results. Thirdly, the role of action planning and plan enactment as post-motivational mediators of the association between intention and behaviour and how the interaction between these determinants could influence the students` condom use were not assessed because these are better assessed with longitudinal studies rather than cross-sectional studies [70]. Finally, the limited number of participants prevented the gender analysis of the data to identify differences between male and female students to develop more gender-sensitive interventions.

## Conclusion

Unprotected sex is common among university students despite the attempts to promote condom use in Sudan. HIV-related knowledge, exposure to condom use cues, attitude towards condom use, peer norms favouring condom use and condom use self-efficacy are all associated with consistent condom use among university students in Sudan. Interventions seeking to promote consistent condom use among sexually active students should increase knowledge about HIV transmission and prevention, address perceived condom disadvantages and foster peer norms favouring condom use. Moreover, such interventions should enhance students` self-efficacy to avoid unprotected sex. Increasing students’ exposure to condom use cues may also help them use condoms consistently.

## Data Availability

The datasets used during the current study are available from the corresponding author on reasonable request.
